# IRNSS/NavIC L5 Attitude Determination

**DOI:** 10.3390/s17020274

**Published:** 2017-01-30

**Authors:** Safoora Zaminpardaz, Peter J.G. Teunissen, Nandakumaran Nadarajah

**Affiliations:** 1 GNSS Research Centre, Department of Spatial Sciences, Curtin University, GPO Box U1987, Perth, WA 6845, Australia; p.teunissen@curtin.edu.au (P.J.G.T.); n.nadarajah@curtin.edu.au (N.N.); 2 Department of Geoscience and Remote Sensing, Delft University of Technology, PO Box 5048, 2600 GA Delft, The Netherlands

**Keywords:** Indian Regional Navigation Satellite System (IRNSS), Navigation with Indian Constellation (NavIC), integer carrier-phase ambiguity resolution, attitude determination, multivariate constrained integer least-squares, MC-LAMBDA

## Abstract

The Indian Regional Navigation Satellite System (IRNSS) has recently (May 2016) become fully-operational and has been provided with the operational name of NavIC (Navigation with Indian Constellation). It has been developed by the Indian Space Research Organization (ISRO) with the objective of offering positioning, navigation and timing (PNT) to the users in its service area. This contribution provides for the first time an assessment of the IRNSS L5-signal capability to achieve instantaneous attitude determination on the basis of data collected in Perth, Australia. Our evaluations are conducted for both a linear array of two antennas and a planar array of three antennas. A pre-requisite for precise and fast IRNSS attitude determination is the successful resolution of the double-differenced (DD) integer carrier-phase ambiguities. In this contribution, we will compare the performances of different such methods, amongst which the unconstrained and the multivariate-constrained LAMBDA method for both linear and planar arrays. It is demonstrated that the instantaneous ambiguity success rates increase from 15% to 90% for the linear array and from 5% to close to 100% for the planar array, thus showing that standalone IRNSS can realize 24-h almost instantaneous precise attitude determination with heading and elevation standard deviations of 0.05° and 0.10°, respectively.

## 1. Introduction

Developed by the Indian Space Research Organization (ISRO), the Indian Regional Navigation Satellite System (IRNSS) has recently (May 2016) become fully-operational and provided with the operational name of NavIC (Navigation with Indian Constellation). This new addition to Global Navigation Satellite Systems (GNSSs) aims to provide positioning, navigation and timing (PNT) to the users in its service area. The IRNSS satellites transmit navigation signals, based on Code Division Multiple Access (CDMA), on L5 (1176.45 MHz) with a Binary Phase-Shift Key (BPSK (1)) modulation for standard positioning service (SPS) users, and with a Binary Offset Carrier (BOC (5,2)) modulation for restricted service (RS) users [1]. The fully-operational IRNSS constellation consists of three geostationary orbit (GEO) satellites and four inclined geosynchronous orbit (IGSO) satellites (see Table 1).

IRNSS real data have hitherto formed the basis of several studies. The clock stability of I1 is assessed in [3], while the accuracy of a precise model for solar radiation pressure is tested using the I1 and I2 observations in [4]. The data of I1, I2 and I3 are used by [5] for comparison of the orbit determination methods, and by [6] to validate the orbit accuracy with modernized ephemeris parameters. The first positioning results based on the data of I1, I2, I3, and I4 over India are presented in [7], and over Australia in [8]. The observations of I1 and I2 were used by [9] to investigate the quality of the IRNSS navigation messages.

In this contribution, for the first time, an analysis of the *fully-operational* IRNSS L5-signal capability to achieve *instantaneous attitude determination* is carried out. Such an analysis based on only *two* IRNSS satellites, i.e., I1 and I2, was conducted by [10]. Using multiple GNSS antennas rigidly mounted on a platform in known formation, one can determine the attitude of this platform, see e.g., [11,12,13,14], which can be a vessel, a land vehicle, an aircraft or a space platform [15,16,17,18,19]. Precise and fast GNSS-based attitude determination would be realized through incorporation of the very precise phase observations, and successful resolution of the double-differenced (DD) integer carrier-phase ambiguities is the key to fully exploit the phase observations. Resulting in the highest possible ambiguity resolution success rate, the Least squares AMBiguity Decorrelation Adjustment (LAMBDA) method developed by [20,21,22] is the standard method used for the unconstrained mixed-integer GNSS observational model. As for the GNSS attitude model, the local antenna geometry is known in the body (platform) frame, which can be exploited to further improve the ambiguity resolution performance. To realize this, the multivariate constrained (MC-)LAMBDA method has been developed [18,19,23,24,25,26,27,28]. This method incorporates the known local antenna geometry in a rigorous manner, leading to higher success rates w.r.t. the standard LAMBDA.

In this study, our evaluations are conducted for both a linear array of two antennas and a planar array of three antennas collecting L5 signals of IRNSS at Curtin University, Perth, Australia. We first explain our GNSS-based single-frequency attitude determination method. The performance of the IRNSS L5 observables for the instantaneous attitude determination is presented for both the linear and planar array. It is also shown what improvements are achieved when using MC-LAMBDA instead of standard LAMBDA method. Finally, a summary and conclusions are given.

## 2. GNSS Observational Model

In this section, the single-frequency GNSS model of observations is described for both the unconstrained and multivariate-constrained scenarios. For the latter, the known body-geometry of the antenna array is taken into account.

### 2.1. Unconstrained Model

Assume that *n* antennas, firmly mounted on a platform, are simultaneously tracking *m* IRNSS satellites on L5 frequency. We further assume that the array is of a small scale such that the differential atmospheric delays (troposphere and ionosphere) and orbital errors between the antennas can be neglected. The multivariate linearized single-epoch DD GNSS array model of observations then reads

(1) $$\begin{array}{ccc} E[\left(D_{m}^{T}⊗D_{n}^{T}\right)\left[\begin{array}{c} \phi \\ p \end{array}\right]] & = & \left[\begin{array}{cc} M & \;A\\ M & \;0 \end{array}\right]\;\left[\begin{array}{c} vec(X^{T})\;\\ a \end{array}\right],\\ D[\left(D_{m}^{T}⊗D_{n}^{T}\right)\left[\begin{array}{c} \phi \\ p \end{array}\right]] & = & \left[\begin{array}{cc} \sigma_{\phi }^{2}\;Q⊗P & \;0\;\\ 0 & \;\sigma_{p}^{2}\;Q⊗P \end{array}\right], \end{array}$$

where E[.], D[.], ⊗ and vec(.) denote the expectation and dispersion operator, Kronecker product and vec-operator [29,30], respectively. The undifferenced *observed-minus-computed* phase and code observations are, respectively, collected in the mn-vectors *ϕ* and *p* with the following structure: y={ϕ,p}, $y={\left[y^{1^{T}},\;y^{2^{T}},...,y^{m^{T}}\right]}^{T}$, with $y^{s}={\left[y_{1}^{s},\;y_{2}^{s},...,y_{n}^{s}\right]}^{T}$ and with $y_{r}^{s}$ being the phase/code observation between antenna *r* and satellite *s*. The (m−1)×m matrix $D_{m}^{T}=\left[-e_{m-1},\;I_{m-1}\right]$ is the differencing matrix forming the between-satellite single-differencing, while the (n−1)×n matrix $D_{n}^{T}=\left[-e_{n-1},\;I_{n-1}\right]$ forms the between-receiver single-differencing. *e* and *I* are, respectively, the vector of ones and the identity matrix of which the dimension is specified by their subscripts. The unknown baseline components in NED (North–East–Down) frame are included in 3×(n−1) matrix *X*, and the unknown integer DD ambiguities, in cycle, in (m−1)(n−1)-vector *a*. Their corresponding design matrices are of the form of $M=D_{m}^{T}G⊗I_{n-1}$ and $A=\lambda \;I_{m-1}⊗I_{n-1}$, where *G* contains the receiver–satellite unit direction vectors as its rows and *λ* is the wavelength of frequency L5.

The stochastic model is formed by the (m−1)×(m−1) matrix $Q=D_{m}^{T}W^{-1}D_{m}$ and (n−1)×(n−1) matrix $P=D_{n}^{T}D_{n}$. $W=\mathrm{diag}(w^{1},\;w^{2},...,\;w^{m})$ is an m×m diagonal matrix of which the diagonal entries $w^{s}\;\left(s=1,...,m\right)$ capture the elevation-dependency of the IRNSS observations and are given as [31]

(2) $$w^{s}\;=\;{\left[1\;+\;10\;\exp\left(-\frac{\theta^{s}}{10}\right)\right]}^{-2},$$

where $\theta^{s}$ is the elevation of the satellite *s* in degrees. The zenith-referenced standard deviation of the undifferenced phase and code observables are denoted as $\sigma_{\phi }$ and $\sigma_{p}$, respectively.

### 2.2. Multivariate-Constrained Model

Taking into account the known antennas’ geometry in the body frame, the model of observations in Equation (1) can be strengthened. The baseline coordinates in the body frame *B* can be transferred to their counterparts in NED frame *X* through [32]

(3) $$X=\;R\;B;\;X,\;B\in R^{3\times (n-1)},\;R\in O^{3\times 3},$$

where *R* is a rotation matrix satisfying $R^{T}R=I_{3}$ and det(R)=+1 [33]. Combining Equations (1) and (3), the following replacements are required

(4) $$vec\left(X^{T}\right)\to vec\left(R^{T}\right);\;D_{m}^{T}G⊗I_{n-1}\to D_{m}^{T}G⊗B^{T}.$$

Since the rotation matrix *R* is of full rank, we have rank(X)=rank(B)=q. The baselines achieve their full span if q=min(3,n−1) [34]. In case q<min(3,n−1), the transpose of the baseline matrix $B^{T}$ forming the design matrix $D_{m}^{T}G⊗B^{T}$ would be rank deficient. In order to rule this case out, we assume that the body frame axes are formed by the first *three* baselines that are represented in the body frame as [32,34]

(5) $$\left[b_{1},\;b_{2},\;b_{3}\right]\;=\;\left[\begin{array}{ccc} b_{11} & b_{21} & b_{31}\\ 0 & b_{22} & b_{32}\\ 0 & 0 & b_{33} \end{array}\right].$$

Therefore, Equation (3) would be replaced by

(6) $$X=\;R_{q}\;B;\;,\;X\in R^{3\times (n-1)},\;B\in R^{q\times (n-1)},\;R_{q}\in O^{3\times q}$$

In the sequel, we will work with Equation (6) instead of Equation (3). Our analyses are based on a linear array of one baseline and a planar array of two baselines, for both of which q=n−1.

### 2.3. Attitude Determination

The aim of the attitude determination is to determine matrix $R_{q}$ in Equation (6) from which (some of) the attitude parameters, i.e., heading (*α*), elevation (*ϵ*) and bank (*β*), can be extracted. As an example, when q=3, $R_{3}$ can be parametrized as

(7) $$R_{3}\;=\;\left[\begin{array}{ccc} c_{\alpha }c_{\epsilon } & \;-s_{\alpha }c_{\beta }+c_{\alpha }s_{\epsilon }s_{\beta } & \;s_{\alpha }s_{\beta }+c_{\alpha }s_{\epsilon }c_{\beta }\\ s_{\alpha }c_{\epsilon } & \;c_{\alpha }c_{\beta }+s_{\alpha }s_{\epsilon }s_{\beta } & \;-c_{\alpha }s_{\beta }+s_{\alpha }s_{\epsilon }c_{\beta }\\ -s_{\epsilon } & \;c_{\epsilon }s_{\beta } & \;c_{\epsilon }c_{\beta } \end{array}\right],$$

in which $c_{\left\{.\right\}}=\cos\left\{.\right\}$ and $s_{\left\{.\right\}}=\sin\left\{.\right\}$.

The least-squares solutions for the orthonormal matrix ${\overset{ˇ}{R}}_{q}$ and the integer vector $\overset{ˇ}{a}$ based on Equations (1) and (4), are obtained through solving the following minimization problems [32]

(8) $$\begin{array}{c} {\overset{ˇ}{R}}_{q}\left(a\right)=\underset{R_{q}\in O^{3\times q}}{\mathrm{argmin}}\;||vec\left({\hat{R}}_{q}\left(a\right)-R_{q}\right){\left|\right|}_{Q_{vec({\hat{R}}_{q}\left(a\right))}}^{2},\\ \overset{ˇ}{a}=\underset{a\in Z^{\left(m-1)\times (n-1\right)}}{\mathrm{argmin}}\;\left(\left|\right|\hat{a}-{a||}_{Q_{\hat{a}\hat{a}}}^{2}+||vec\left({\hat{R}}_{q}\left(a\right)-{\overset{ˇ}{R}}_{q}\left(a\right)\right){\left|\right|}_{Q_{vec({\hat{R}}_{q}\left(a\right))}}^{2}\right),\\ {\overset{ˇ}{R}}_{q}={\overset{ˇ}{R}}_{q}\left(\overset{ˇ}{a}\right), \end{array}$$

where $vec\left({\hat{R}}_{q}\left(a\right)\right)=vec\left({\hat{R}}_{q}\right)-Q_{vec\left({\hat{R}}_{q}\right)\hat{a}}Q_{\hat{a}\hat{a}}^{-1}\left(\hat{a}-a\right)$. ${\hat{R}}_{q}$ and $\hat{a}$ are the least-squares solutions disregarding the orthonormality of the rotation matrix and integerness of the DD ambiguities, and $Q_{vec({\hat{R}}_{q})}$, $Q_{\hat{a}\hat{a}}$ and $Q_{vec\left({\hat{R}}_{q}\right)\hat{a}}$ are their corresponding variance and covariance matrices. The expression to be minimized in the second minimization problem of Equation (8) is the ambiguity objective function, which is nonstandard due to the presence of the second term. To solve this minimization problem, the MC-LAMBDA method has been developed [18,19,23,24,25,26,27,28,35], incorporating the orthonormality of the rotation matrix in a rigorous manner. This method therefore leads to higher success rates w.r.t. the standard LAMBDA which only takes into account the integerness of the DD ambiguities. In this contribution, the performance of both LAMBDA and MC-LAMBDA is investigated.

As was mentioned, our analyses are based on a linear array of one baseline and a planar array of two baselines satisfying q=n−1. For such a situation, matrix *B* would become invertible, and Equation (8) can alternatively be written as [34]

(9) $$\begin{array}{c} \overset{ˇ}{X}\left(a\right)=\underset{X^{T}X=B^{T}B}{\mathrm{argmin}}\;||vec\left(\hat{X}\left(a\right)-X\right){\left|\right|}_{Q_{vec(\hat{X}\left(a\right))}}^{2},\\ \overset{ˇ}{a}=\underset{a\in Z^{\left(m-1)\times (n-1\right)}}{\mathrm{argmin}}\;\left(\left|\right|\hat{a}-{a||}_{Q_{\hat{a}\hat{a}}}^{2}+||vec\left(\hat{X}\left(a\right)-\overset{ˇ}{X}\left(a\right)\right){\left|\right|}_{Q_{vec(\hat{X}\left(a\right))}}^{2}\right),\\ \overset{ˇ}{X}=\overset{ˇ}{X}\left(\overset{ˇ}{a}\right). \end{array}$$

Therefore, in a single-baseline scenario, the constraint in the first expression of Equation (9) is a constraint on the baseline length, i.e., ||x||=l. For such a situation, $\overset{ˇ}{x}\left(a\right)$ is a vector on the sphere of radius *l* that has the smallest distance to $\hat{x}\left(a\right)$, where distance is measured with respect to the metric as defined by the variance matrix $Q_{\hat{x}\left(a\right)\hat{x}\left(a\right)}$ [35].

## 3. Numerical Analysis

In this section, we present our numerical analysis of IRNSS L5 attitude determination performance.

### 3.1. Measurement Set-Up

**Figure 1:**
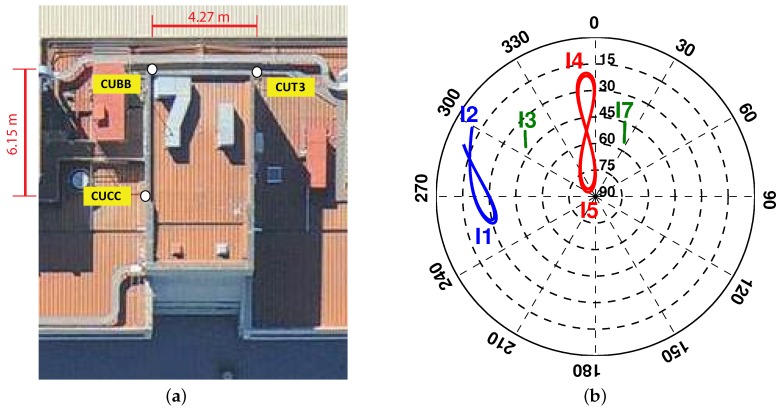
(**a**) Stations CUCC, CUBB and CUT3 at Curtin University equipped with JAVAD TRE_G3TH_8 receivers, connected to TRM59800.00 SCIS antennas; (**b**) 24-h IRNSS/NavIC skyplot at Perth on DOY 166 of 2016 with the cut-off elevation of $10^{\circ }$.

Our evaluations in this study are on the basis of data taken from three stations, namely CUCC, CUBB and CUT3 of short baselines at Curtin University, Perth, Australia (Figure 1a). Each station is equipped with a JAVAD TRE_G3TH_8 receiver and connected to a TRM59800.00 SCIS antenna. The data-set contains the 1-second IRNSS L5 observations collected with a cut-off elevation angle of $10^{\circ }$ on DOY (Day Of Year) 166 of 2016. Our analyses are conducted on an epoch-by-epoch basis, using the broadcast ephemeris. Figure 1b illustrates the 24-h skyplot of IRNSS at Perth. Prior to our analyses, we need to consider representative values for the zenith-referenced standard deviations in Equation (1), i.e., $\left\{\sigma_{\phi },\;\sigma_{p}\right\}$. Applying the least-squares variance component estimation (LS-VCE) [36] to the 1-s data of DOYs 155 and 157 of 2016, the mentioned standard deviations were estimated as $\sigma_{\phi }=2$ mm and $\sigma_{p}=26$ cm.

### 3.2. Baseline Solution: From Unconstrained to Constrained

**Figure 2:**
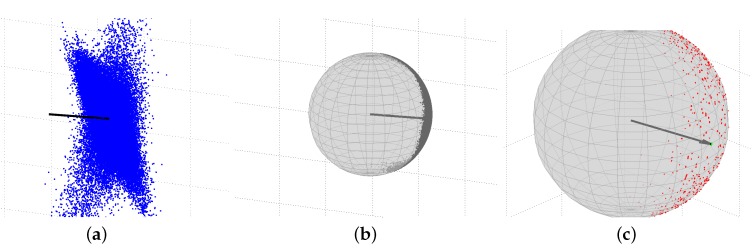
Single-epoch IRNSS L5 solutions of the CUCC–CUBB baseline at Curtin University on DOY 166 of 2016 with the cut-off elevation of $10^{\circ }$. (**a**) unconstrained ambiguity–float scenario; (**b**) constrained (||x||=l) ambiguity–float scenario using MC-LAMBDA (multivariate-constrained LAMBDA); (**c**) constrained (||x||=l) ambiguity–fixed scenario using MC-LAMBDA. The vector shown in all the three panels denotes the baseline ground truth. The sphere in panels (**b**) and (**c**) is zero-centered with a radius of the CUCC–CUBB baseline length. In panel (**c**), green and red dots show the correctly-fixed and wrongly-fixed solutions, respectively.

In order to assess the IRNSS L5 attitude determination performance, we first consider a linear array formed by the antenna pair CUCC–CUBB (see Figure 1). Figure 2 for this baseline demonstrates how the constraint on the baseline length affects the baseline solutions. Shown in Figure 2a is a zoom-in of the single-epoch IRNSS L5 solutions (blue dots) for the unconstrained ambiguity–float scenario, as well as the baseline ground truth (black vector). The dispersion in the baseline solutions is governed by the code precision and satellites geometry. The excursions in this three-dimensional scatter plot are due to the significant change that the receiver-satellite geometry undergoes during a 24-h period. Upon constraining the baseline length with e.g., ||x||=l, the corresponding solutions can only vary on a sphere with the radius of *l*.

Figure 2b illustrates the single-epoch IRNSS L5 solutions for the constrained (||x||=l) ambiguity–float scenario (gray dots), the baseline ground truth (gray vector) and the sphere with the radius of *l*. As it can be seen, the baseline solutions all lie on the shown sphere. Resolving the integer DD ambiguities, Figure 2c shows the single-epoch IRNSS L5 solutions for the constrained (||x||=l) ambiguity–fixed scenario (green dots: correctly-fixed; red dots: wrongly-fixed), the baseline ground truth (gray vector) and the sphere with the radius of *l*. In order to have a better view of the correctly-fixed solutions, this panel has been rotated with respect to the first two panels. For this scenario, there are different clusters of the baseline solutions that correspond to different estimated integer values for the DD ambiguities. The green cluster associates with the correct integer value comprising 89.2% of the fixed solutions, while the red clusters correspond to the wrong integer values. To judge whether a DD ambiguity is correctly fixed, its corresponding integer solution is compared with the reference integer DD ambiguity computed based on the multi-epoch solution of the baseline-known model.

**Figure 3:**
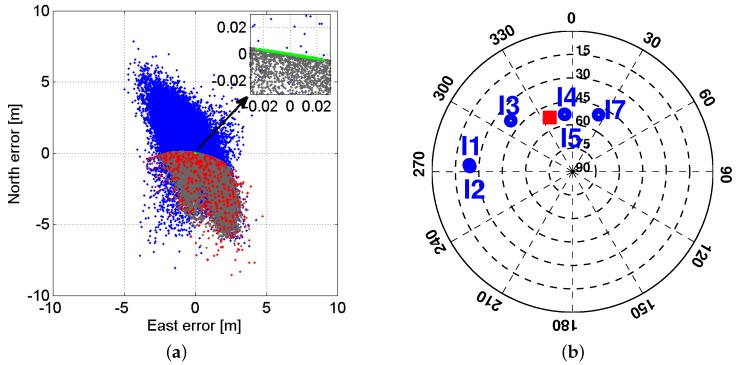
(**a**) Single-epoch IRNSS L5 solutions of the CUCC–CUBB baseline at Curtin University corrected for the ground truth, on DOY 166 of 2016 with the cut-off elevation of $10^{\circ }$. blue: unconstrained ambiguity–float solutions; gray: constrained (||x||=l) ambiguity–float solutions; red: constrained (||x||=l) wrongly-fixed solutions using MC-LAMBDA; green: constrained (||x||=l) correctly-fixed solutions using MC-LAMBDA. A zoom-in is also depicted in the upper-right of the figure; (**b**) Day-averaged IRNSS skyplot at Perth for DOY 166 of 2016 with the cut-off elevation of $10^{\circ }$. The red square indicates the skyplot position of vector $\bar{u}$ (cf. Equation (11)).

Shown in Figure 3a is the horizontal scatter plot of the single-epoch IRNSS L5 solutions for all the scenarios depicted in Figure 2, corrected for the baseline ground truth. Note that the blue scatter plot is elongated in an almost North–Westerly direction, which can be explained by means of the receiver-satellite geometry. Since confidence ellipse is the formal representative of the empirical scatter plot, we concentrate on the confidence ellipse. Denoting the unconstrained ambiguity–float baseline solution as $\hat{x}$ with mean and covariance matrix of, respectively, *x* and $Q_{\hat{x}\hat{x}}$, its confidence ellipse reads

(10) $${\left(\hat{x}-x\right)}^{T}Q_{\hat{x}\hat{x}}^{-1}\left(\hat{x}-x\right)=k^{2},$$

in which the constant $k^{2}$ is chosen such that a certain confidence level is reached. As the direction of elongation is given by the direction of the eigenvector of $Q_{\hat{x}\hat{x}}^{-1}$ corresponding to its smallest eigenvalue, it follows with the aid of Equation (1) that this direction is given by

(11) $$\begin{array}{ccc} f\; & = & \;\underset{\tilde{f}}{\mathrm{argmin}}\;{\tilde{f}}^{T}Q_{\hat{b}\hat{b}}^{-1}\tilde{f}\\ & = & \;\underset{\tilde{f}}{\mathrm{argmin}}\sum_{s=1}^{m}w^{s}{\left[{\tilde{f}}^{T}\left(u^{s}-\bar{u}\right)\right]}^{2}, \end{array}$$

with $u^{s}$ being the unit direction vector from receiver to satellite *s*, and $\bar{u}$ being the weighted average of the vectors $u^{s}\;\left(s=1,...,m\right)$. Figure 3b depicts the day-averaged skyplot position of the IRNSS satellites as well as that of the weighted-average at Perth on DOY 166 of 2016 with the cut-off elevation of $10^{\circ }$. As the differences are mainly oriented along the North–East direction, the direction *f* that minimizes their contribution to Equation (11) will mainly lie in a North–Westerly direction.

Now, we turn our focus onto the constrained scenario. The gray dots in Figure 3a show the horizontal scatter plot of the constrained ambiguity–float baseline solutions, while the red and green dots show that of the constrained correctly- and wrongly-fixed baseline solutions. A zoom-in is also provided in the upper-right of the figure to show the correctly-fixed results more clearly. As it can be seen, while the ambiguity–float results are biased, the correctly-fixed results are unbiased. This can be attributed to the precision of the contributing observations and the nonlinearity of the baseline length constraint. For the single-epoch ambiguity–float scenario, the precision of the baseline solution is only dependent on the less precise code observables, whereas, for the ambiguity–fixed scenario, it are the very precise phase observations that play the leading role in the baseline estimation. In the following, we give a two-dimensional example to elaborate how the poor precision of the observations may lead to a bias in the constrained baseline solution.

**Figure 4:**
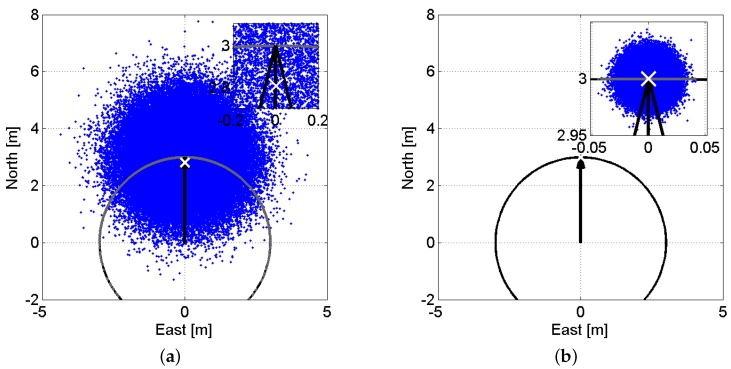
Visualization of the baseline-length constraint nonlinearity as function of data precision. Blue dots are samples of a two-dimensional baseline simulated from a normal distribution with the mean of $b={\left[3\;0\right]}^{T}$ m and variance matrix of $Q=\sigma^{2}\;I_{2}$. Gray dots show the baseline-length-constrained counterparts (||b||=3 m) of blue dots. The black vector indicates the baseline ground truth position and the black circle is centred at $b={\left[0\;0\right]}^{T}$ with the radius of 3 m. The white cross shows the mean value of the gray scatter plot. In each panel, a zoom-in is also provided in the upper-right of the panel. (**a**) σ=1 m; (**b**) σ=0.01 m.

Suppose that we have a two-dimensional baseline with the length of l=3 m and azimuth of $\alpha =0^{\circ }$. The corresponding baseline North–East coordinates then read $b={\left[3\;0\right]}^{T}$ m. By the use of normal distribution, we simulate $10^{5}$ samples of baseline north and east components with the mean of $b={\left[3\;0\right]}^{T}$ m and the variance matrix of $Q=I_{2}$ (identity matrix). These samples are shown as blue dots in Figure 4a. In this figure, the true position of the baseline is shown as the black vector. Now, if we impose a constraint on the baseline length, the blue dots are mapped onto a circle with the radius equal to the baseline length l=3 m. This circle is also shown in black in Figure 4a. Due to the poor precision of the baseline samples, they are mapped onto a large part of the circle circumference.

The non-negligible curvature of the area onto which the samples are mapped makes the constrained solutions biased. The mean value of the constrained solutions is depicted as the white cross. This bias is called the *nonlinearity bias* [37]. Now, we switch to a second scenario where the precision of the samples are 100 times better, i.e., $Q=10^{-4}\;I_{2}$. Figure 4b illustrates the counterparts of Figure 4a for $Q=10^{-4}\;I_{2}$. For this scenario, due to the very small variability of the simulated samples, they are mapped, upon constraining the baseline length, onto a very small part of the circle circumference, which, as the figure shows, can be considered a straight line. Therefore, the nonlinearity bias for this scenario becomes negligible.

### 3.3. Attitude Determination Performance

In this section, we analyse the IRNSS L5 single-epoch attitude determination performance for the linear array of CUCC–CUBB and the planar array formed by CUCC, CUBB and CUT3 (see Figure 1). The body frame coordinate matrix *B* for the planar array is given by

$$B=\left[\begin{array}{cc} 6.15 & 6.78\\ 0 & 4.22 \end{array}\right].$$

Table 2, for the mentioned linear and planar arrays, presents the IRNSS L5 single-epoch empirical and formal standard deviations of the attitude angles for both the ambiguity–float and ambiguity–fixed scenarios. The ambiguity–fixed solutions are obtained through applying MC-LAMBDA. Formal values are obtained from taking the average of all the single-epoch *linearized* formal least-squares standard deviations, whereas the empirical values are obtained from the single-epoch least-squares estimations of the attitude angles. In the case of the planar array, in addition to heading and elevation, bank is also estimable. Upon fixing the DD ambiguities, the attitude angles improve in precision by almost a factor of 120.

According to this table, in contrast to the ambiguity–fixed results, ambiguity–float outcomes show inconsistency between (linearized) formal and empirical values. This can be attributed to the nonlinearity of the model of observations. The formal standard deviations in Table 2 are obtained through the linear approximation of the model of observations w.r.t. the attitude angles and then applying the error propagation law. Now, by means of Figure 4, we explain how well this linear approximation can describe the uncertainty of the constrained solutions of the baseline and the attitude angles. Applying a linear approximation around $b={\left[3\;0\right]}^{T}$ m in Figure 4 would map all the blue dots onto a line, which touches the shown circle at $b={\left[3\;0\right]}^{T}$ m. In that case, the constrained baseline uncertainty in the North direction would be zero. This indeed can well describe the uncertainty of the constrained baseline in Figure 4b, where the precision of the simulated samples is very high. However, for Figure 4a with samples of poor precision, the constrained baseline uncertainty in the North direction is far larger than zero due to the non-negligible curvature of the area onto which the samples are mapped. This can also explain the inconsistency between the empirical and the (linearized) formal results of the ambiguity–float scenario, where the solutions are achieved on the basis of less precise code observations.

Denoting the standard deviations of heading, elevation and bank by, respectively, $\sigma_{\hat{\alpha }},\;\sigma_{\hat{\epsilon }},\mathrm{and}\;\sigma_{\hat{\beta }}$, Table 2 shows that $\sigma_{\hat{\alpha }}\leq \sigma_{\hat{\epsilon }}\leq \sigma_{\hat{\beta }}$. This can be explained through the baselines orientation along with the IRNSS satellites geometry. As an example, here we consider the linear array of the single-baseline CUCC–CUBB. By the aid of linear approximation, the heading–elevation covariance matrix is given as

(12) $$Q_{\hat{\gamma }\hat{\gamma }}=\frac{\sigma_{p}^{2}}{l^{2}}{\left(\sum_{s=1}^{m}w^{s}\left[J^{T}\left(u^{s}-\bar{u}\right)\right]{\left[J^{T}\left(u^{s}-\bar{u}\right)\right]}^{T}\right)}^{-1}$$

with $\gamma ={\left[\alpha \;\epsilon \right]}^{T}$ and *J* being the Jacobian matrix of the following form

(13) $$J\;=\;\left[J_{\alpha }\;J_{\epsilon }\right]\;=\;\left[\begin{array}{cc} -s_{\alpha }c_{\epsilon } & \;-c_{\alpha }s_{\epsilon }\\ c_{\alpha }c_{\epsilon } & \;-s_{\alpha }s_{\epsilon }\\ 0 & \;-c_{\epsilon } \end{array}\right].$$

**Figure 5:**
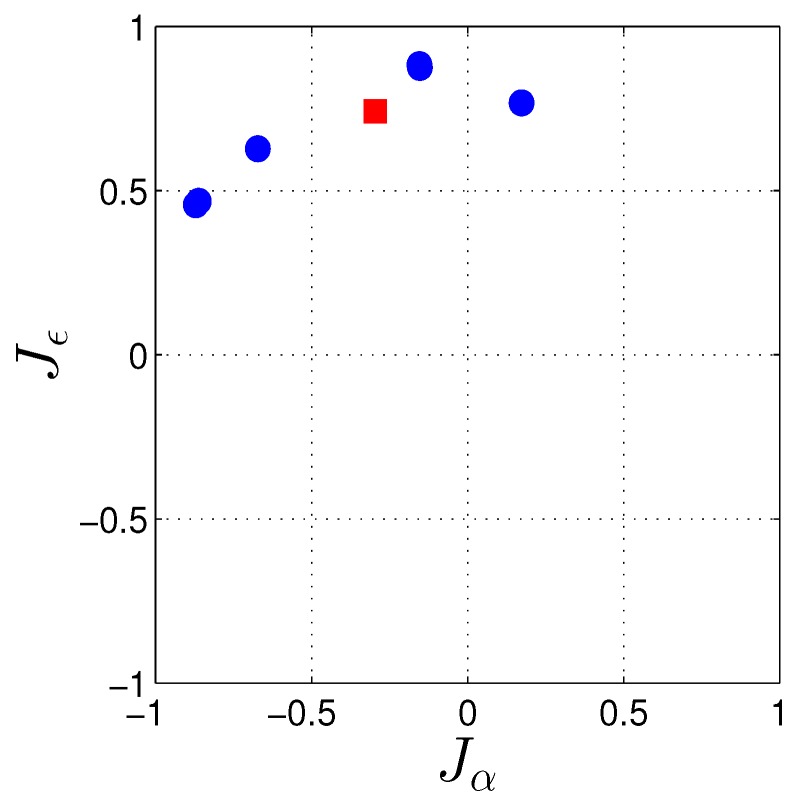
The projection of the day-averaged IRNSS satellites unit direction vectors at Perth onto the plane spanned by $J_{\alpha }\approx {\left[0,\;1,\;0\right]}^{T}$ and $J_{\epsilon }\approx {\left[0,\;\;0,\;-1\right]}^{T}$ (cf. Equation (13)) for DOY 166 of 2016 with the cut-off elevation of $10^{\circ }$. The red square indicates the position of vector $\bar{u}$ projected on $J_{\alpha }$ and $J_{\epsilon }$ (cf. Equations (11) and (12)).

From Equations (12) and (13), if $J_{\alpha }^{T}\left(u^{s}-\bar{u}\right)$ is larger than $J_{\epsilon }^{T}\left(u^{s}-\bar{u}\right)$, then the heading estimation would be more precise than the elevation and vice versa. For the CUCC–CUBB baseline with almost the South–North orientation, we have $J_{\alpha }\approx {\left[0,\;1,\;0\right]}^{T}$ (East direction) and $J_{\epsilon }\approx {\left[0,\;\;0,\;-1\right]}^{T}$ (Up direction). Figure 5 depicts the projection of the day-averaged $u^{s}\;\left(s=1,...,m\right)$ and $\bar{u}$ onto the plane spanned by $J_{\alpha }$ and $J_{\epsilon }$. As the differences $\left(u^{s}-\bar{u}\right)$ have larger projections onto $J_{\alpha }$ w.r.t. $J_{\epsilon }$, heading is expected to have better precision than elevation. Equation (12) in addition reveals that the longer the baseline, the more precise the attitude angles estimations.

Our ambiguity–fixed results are in good consistency with the GPS L1-based ones presented in [38]. There, an almost South–North oriented 8-meter baseline is used and the phase standard deviation is considered to be 1 mm. Given that the CUCC–CUBB baseline has almost the South–North orientation and the length of almost 6 m, and also that the IRNSS L5 phase precision is 2 mm, it is expected that the attitude precision in [38] be better by a factor of $\frac{8}{3}$ compared to those listed in Table 2. Such superiority is indeed confirmed by the presented results.

### 3.4. Ambiguity Resolution Performance

**Figure 6:**
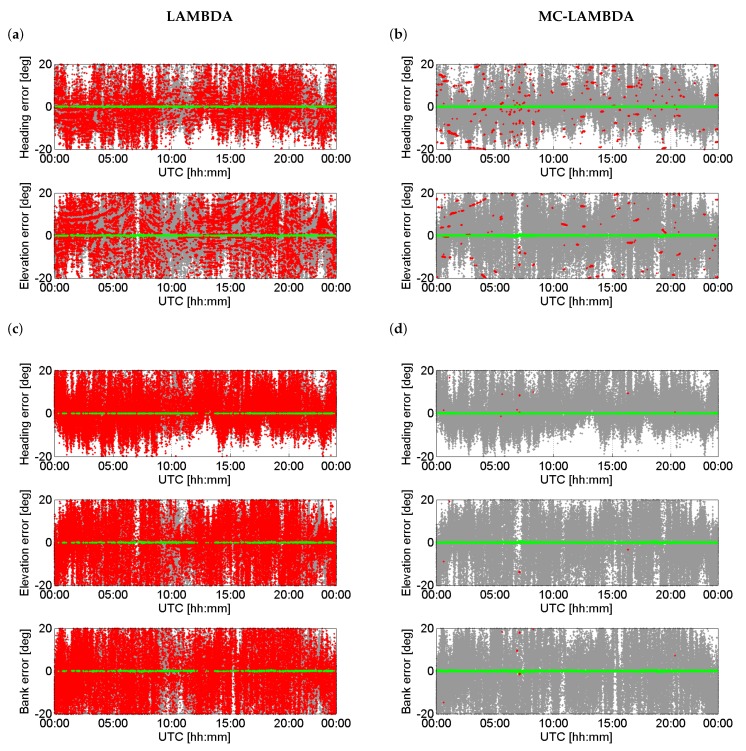
Time series of the IRNSS L5 single-epoch solutions for the attitude angles based on the data collected on DOY 166 of 2016 at Perth with the cut-off elevation of $10^{\circ }$. Each panel shows three types of solutions: gray—ambiguity–float solutions; red—wrongly-fixed solutions; green—correctly-fixed solutions. The fixed solutions on the left are estimated through LAMBDA, while those on the right are estimated through MC-LAMBDA. (**a**,**b**) correspond to the linear array formed by CUCC–CUBB; (**c**,**d**) correspond to the planar array formed by CUCC–CUBB–CUT3.

Having investigated the IRNSS L5 attitude determination performance, we now concentrate on the ambiguity resolution performance. To do so, we consider both the aforementioned linear and planar arrays and make use of both the standard LAMBDA and MC-LAMBDA method. As was explained in Introduction, MC-LAMBDA was developed for attitude determination and is advantageous over LAMBDA due to the inclusion of the rotation matrix orthonormality. Figure 6 shows the 24-h time series of the IRNSS L5 single-epoch solutions for the attitude angles for linear array of CUCC–CUBB (a and b) and planar array of CUCC–CUBB–CUT3 (c and d). The fixed solutions on the left are estimated through LAMBDA, while those on the right are estimated through MC-LAMBDA.

From Figure 6, switching from LAMBDA to MC-LAMBDA, the number of wrongly-fixed solutions decreases dramatically. The percentage of correctly fixed solutions, known as the ambiguity resolution success rate, increases from 14.6% to 89.2% for the linear array, and from 4.8% to 99.8% for the planar array. Higher success rates would be achievable if we include the data of more than one epoch. Since the MC-LAMBDA success rate is already large, only a few number of epochs are needed to achieve higher success rates. This indicates that, upon using MC-LAMBDA, standalone IRNSS can realize 24-h almost instantaneous precise attitude determination.

From linear array to planar array, while the ambiguity success rate of LAMBDA decreases, that of MC-LAMBDA increases. In the case of MC-LAMBDA, which takes into account the constraint $X^{T}X=B^{T}B$, the model gets stronger from linear array to planar array due to the inclusion of a larger number of constraints, which, in turn, leads to ambiguity resolution improvements. The LAMBDA-based success rate Ps for single-frequency DD ambiguities, corresponding with *n* antennas and *m* satellites, can be well approximated by [39,40]

(14) $$Ps\;\approx \;{\left[2\Phi \left(\frac{1}{\mathrm{ADOP}}\right)-1\right]}^{\left(m-1)(n-1\right)},$$

where $\Phi \left(x\right)=\int_{-\infty }^{x}\frac{1}{\sqrt{2\pi }}\exp\left\{-\frac{1}{2}v^{2}\right\}dv$. ADOP (Ambiguity Dilution Of Precision) was introduced in [21], defined as the square root of the determinant of the ambiguity variance matrix raised to the power of one over the ambiguity dimension. Considering the model of observations in Equation (1), it can be shown that the ADOP corresponding with n=2 is only 1.07 times larger than the ADOP corresponding with n=3. Therefore, the ADOP of n=2 is almost the same as the ADOP of n=3. For a given value of ADOP, the success rate in Equation (14) decreases as *n* increases. Thus, the LAMBDA-based success rate is indeed expected to decrease as one switches from the linear array (n=2) to the planar array (n=3).

## 4. Conclusions

In this contribution, we provided an initial assessment of the *fully-operational* IRNSS L5-signal capability to achieve the *instantaneous attitude determination*. We first studied the noise characteristics of the IRNSS L5-signal through the LS-VCE method, and estimated the code and phase zenith-referenced standard deviations as 26 cm and 2 mm, respectively. Our evaluations of IRNSS attitude determination performance were conducted for both a linear array of two antennas and a planar array of three antennas located at Curtin University, Perth, Australia. For a linear array, we showed schematically in a stepwise manner how the inclusion of the baseline length constraint and then the integerness of the DD ambiguities affect the baseline least-squares solutions.

It was shown that, for the single-epoch constrained ambiguity–float scenario, where only the less precise code observations contribute to the baseline estimation, due to the nonlinearity of the baseline length constraint and poor precision of the observations, a large bias, called nonlinearity bias, affects the solution of the attitude angles and hence the constrained baseline solutions. However, if one takes into account the integerness of the DD ambiguities, the very precise phase observations will play the leading role in estimation of attitude angles, thus making the corresponding bias negligible.

A pre-requisite for precise attitude determination is the successful resolution of the DD integer ambiguities. The performance of LAMBDA and MC-LAMBDA was compared for both the linear and planar array. The ambiguity success rate was shown to increase from 14.6% to 89.2% for the linear array, and from 4.8% to 99.8% for the planar array. Higher success rates would be achievable if we include the data of more than one epoch. Since the MC-LAMBDA success rate is already large, only a small number of epochs are needed to achieve higher success rates. This indicates that, upon using MC-LAMBDA, standalone IRNSS can realize 24-h almost instantaneous precise attitude determination. Upon fixing the integer ambiguities, it was shown for the linear array oriented in the South–North direction, with the length of around l=6 m, that heading and elevation are estimable with the standard deviations of 0.05° and 0.10°. The higher precision of heading compared to the elevation was explained through the baseline orientation and IRNSS satellites geometry. Since IRNSS satellites are mainly located in the North–West quadrant of the Perth skyplot, the mentioned precisions are also obtained if the baseline is oriented in the East–West direction. This is also confirmed by the results we obtained using real data.

## Figures and Tables

**Table 1:** Information on the IRNSS/NavIC satellites [2].

Satellite	Type	Longitude	Inclination	Launch Date
IRNSS-1A (I1)	IGSO	$55^{\circ }$ E	$29.0^{\circ }$	July 2013
IRNSS-1B (I2)	IGSO	$55^{\circ }$ E	$31.0^{\circ }$	April 2014
IRNSS-1C (I3)	GEO	$83^{\circ }$ E	–	October 2014
IRNSS-1D (I4)	IGSO	$111.75^{\circ }$ E	$30.5^{\circ }$	March 2015
IRNSS-1E (I5)	IGSO	$111.75^{\circ }$ E	$28.1^{\circ }$	January 2016
IRNSS-1F (I6)	GEO	$32.5^{\circ }$ E	–	March 2016
IRNSS-1G (I7)	GEO	$129.5^{\circ }$ E	–	April 2016

**Table 2:** IRNSS L5 single-epoch empirical and linearized formal standard deviations of the attitude angles for the linear array of CUBB–CUCC and the planar array of CUBB–CUCC–CUT3 (see Figure 1), for both the ambiguity–float and ambiguity–fixed scenarios based on the data collected on DOY 166 of 2016 with the cut-off elevation angle of $10^{\circ }$. The ambiguity–fixed solutions are obtained through applying MC-LAMBDA (multivariate-constrained LAMBDA). emp: empirical; form: formal; STD: standard deviation.

Scenario	Ambiguity-Float				Ambiguity-Fixed			
	Linear Array		Planar Array		Linear Array		Planar Array	
	Emp	Form	Emp	Form	Emp	Form	Emp	Form
heading STD [deg]	13.36	7.27	12.66	6.33	0.04	0.05	0.04	0.05
elevation STD [deg]	19.73	17.91	20.41	16.46	0.09	0.14	0.09	0.13
bank STD [deg]	–	–	25.99	21.21	–	–	0.11	0.16
